# Asymptomatic central line-associated bloodstream infections in children implanted with long term indwelling central venous catheters in a teaching hospital, Sri Lanka

**DOI:** 10.1186/s12879-020-05190-5

**Published:** 2020-06-29

**Authors:** J. A. A. S. Jayaweera, D. Sivakumar

**Affiliations:** 1 Department of Microbiology, Faculty of Medicine and Allied Sciences, Rajarata University of Sri Lanka, Microbiology, Saliyapura, Sri Lanka; 2 Teaching Hospital Kandy, Kandy, Sri Lanka

**Keywords:** Healthcare-associated infections, Central line-associated bloodstream infection, Asymptomatic bacteremia, *Staphylococcus aureus*, MRSA, Coagulase-negative *Staphylococcus* sp., And right sided-infective endocarditis

## Abstract

**Background:**

Indwelling central venous catheters (CVC) are used to provide long term hemodialysis. The commonest and the severe complication of CVC is the central line-associated bloodstream infection (CLABSI). This study was done to assess the etiology and infectious complications of CVC in children on long term hemodialysis.

**Methods:**

Children newly undergoing hemodialysis and having indwelling CVC were included. They were followed up to a period of 2-years to assess infectious complications. Catheter bundle care approach was employed to prevent infections and other complications. Automated culture from the central catheter and peripheral vein and 2D echocardiography were done in each hemodialysis. Serial procalcitonin (PCT) was measured. Differential time of positivity (DTP) was used to detect CLABSI. During homestay in weekly telephone conversations were done to assess features of infection, and whenever having, we have asked to admit to the tertiary care unit. Logistic regression was performed, and the significant outcome variable was considered following multivariable analysis as a risk factor.

**Results:**

Blood cultures were positive in 1090 (74.5%) out of 1462 children. According to DTP, 410 (28%) were having CLABSI, while 520 (35.6%) were having bacteremia without CLABSI. Out of 410 CLABSI patients, 79 (19.2%) were asymptomatic. Coagulase-negative *Staphylococcus* spp. (CoNS) bacteremia was significantly associated with asymptomatic CLABSI. Right-sided infective endocarditis (RS-IE) was significantly associated with asymptomatic CLABSI and asymptomatic bacteremia without CLABSI. CoNS was associated significantly in RS-IE following asymptomatic CLABSI and asymptomatic bacteremia. PCT was in asymptomatic CLABSI was 1.8 ± 0.9 ng/mL while in symptomatic CLABSI was 11.3 ± 2.5 ng/ml (*P* = 0.02). CoNS bloodstream infection, tunneled CVC, peripherally inserted central catheter, femoral site, the number of line days > 90, receipt of vancomycin, meropenem, or linezolid in the 5 days before CLABSI diagnosis and recurrent bacteremia were risk factors for asymptomatic CLABSI.

**Conclusions:**

Asymptomatic CLABSI could be a rare occurrence. CoNS was predominantly isolated in patients with asymptomatic CLABSI. RS- IE is a well-known complication in long term indwelling CVC. CoNS was significantly associated with RS-IE following asymptomatic CLABSI. Regular procalcitonin, microbiological, and imaging studies would be essential to detect infectious complications in both symptomatic and asymptomatic patients implanted with long term indwelling CVCs.

## Background

Long-term central venous catheter (CVC) is an invasive device that used in children with chronic renal failure. The device resides in a large central vein, usually the superior vena cava. CVC is used for the administration of fluids, medications, blood products, collection of blood, and hemodialysis (HD) [1]. The most common and severe complication associated with CVC is central line-associated bloodstream infection (CLABSI) [1, 2].

CLABSI rates vary widely, and infection rates depend on device type and patient population [2]. A study conducted in a pediatric intensive care unit (PICU) in United State of America (USA) from 2006 to 2007 reported that incidence of CLABSI was 3.1 per 1000 central line-days [3]. Another study revealed that incidence was 4.1 per 1.000 central line-days in third world countries [4]. Children with haemopoietic stem cell transplantation, prevalence of CLABSI was 5.3 per 1000 central-line days and most commonly identified organism was *Staphylococcus epidermidis* [5].

The etiology and the incidence of Chronic Kidney Disease (CKD) could vary with the age [6]. The prevalence of CKD stage II or lower in children is approximately 18.5–58.3 per million [7]. Compared to adults, in children CKD prevalence is much less but underreporting would mask the true prevalence [8]. In children, structural defects and obstructive uropathy are common before age 5 while hereditary and acquired kidney diseases are common in 5 to 15 years. CKD requires therapeutic measures [9]. When the glomerular filtration rate is below 15 ml/min/1.73 m^2^, renal replacement therapy such as peritoneal dialysis, hemodialysis, or kidney transplantation is indicated [10].

Bacteremia can occur spontaneously following tissue infection, wound care, surgical procedure, and the use of indwelling intravascular catheters [11]. Bacteremia can be symptomatic or asymptomatic. Bacteremia would lead to develop deep seated abscesses, pneumonia, meningitis, and infective endocarditis especially in patients with valvular heart abnormalities [12]. Transient bacteremia is often asymptomatic, but occasionally can develop fever [13]. The development of symptoms usually suggests more severe infections and risk for sepsis or septic shock would be high [11, 14]. CLABSI often leads to continuous bacteremia. This study was conducted to assess the etiology and infectious complications of central line-associated bloodstream infections (CLABSI) in children on long term HD.

## Methods

This was a follow up study. The study was conducted at a pediatric unit in a tertiary care hospital, Sri Lanka, from January-2014 to December-2016. Children (2 years to 12 years) who are newly undergoing hemodialysis and having an indwelling central venous line were included and followed up. They were followed up to 2 years to assess any of the infectious complications. Since in all the patients HD was newly initiated, they were kept > 48 h in the unit. Once patient becomes stable (creatinine decline and reaches a steady level) and no sign of infection they were discharged. We have screened occurrence of CLABSI following after 48 h of insertion of CVC.

Catheter bundle care approach was employed at the time of insertion and maintenance to prevent infections and other complications [15]. In each time before initiating HD, blood (includes asymptomatic patients) was taken from the central line and peripheral site for culture and antimicrobial susceptibility testing (AST). Also, when the patient presented with fever, blood was taken from the central line and peripheral site for culture and antimicrobial susceptibility testing (AST). Every occasion, the same volume of blood was collected under sterile conditions, and BACTEC semi-automated flat form was used for culture. Differential time positivity (DTP) was used to detect CLABSI, bacteremia, central line colonization, and contamination [15, 16]. At day 3 (72 h following initial blood culture), a peripheral blood culture was done to assess the clearance following *Staphylococcus aureus* bacteremia.

Gram staining was performed from all flagged positive BACTEC bottles and inoculated onto blood (10% CO_2_), MacConkey (room air), and chocolate agar (10% CO_2_) to detect the microbial etiology. The inoculated plates were incubated at 35 °C in 10% CO_2_ to enable bacterial colonies to develop [17]. Positive culture isolates were identified using appropriate identification methods, including morphology of colonies, Gram-stain, and an in-house set of biochemical tests and further confirmed using Rapid 20 E (Enterobacteriaceae), NE (Non-Enterobacteriaceae) and S (*Staphylococcus*) semi-automated identification system.

Serial procalcitonin (PCT) and C-reactive protein (CRP) were done to assess the clinical status and the response to the antimicrobial treatment. Also, 2D echocardiography was done in every patient, and in patients with endocarditis serially, it was done to assess the progress/ clinical response. During homestay in weekly telephone conversations were done to assess infectious complications and, if present, asked to admit as soon as possible to the tertiary care facility.

### Definitions

#### CLABSI

The Centers for Disease Control and Prevention (CDC) definitions were used to diagnose CLABSI [18].

### Bacteremia without line infection

The quantitative blood cultures were obtained from the CVC and peripheral vein in same time and isolation of the same pathogen from both cultures with time to positive culture (< 2 h) in the CVC and peripheral sample [15, 16].

### Central line colonizer

Instances where CVC blood culture is positive but the percutaneous blood culture remained negative indicate colonization of the catheter rather than CRBSI. This is especially applicable to organisms like gram-negative rod or enterococcus [15, 16].

### Asymptomatic bacteremia

Instances where the two peripheral blood culture were remained positive for same organism but subject remains without having fever or any other sign/symptom [17].

### Exit site infection

Signs of inflammation is limited to catheter exit site (typically < 2 cm) and having a wound swab/ wound secretion culture positivity [17, 18].

### Tunnel infection

Inflammation extending more than 2 cm from exit site and associated with pain and tenderness along the subcutaneous track and having a wound swab/ wound secretion culture positivity [18].

### Antimicrobial susceptibility testing

The antimicrobial susceptibility testing was performed by the disc diffusion test based on Clinical and Laboratory Standards Institute (CLSI) guidelines (M100s27) [19]. The following antimicrobial agents were tested: ampicillin (30 μg), amikacin (30 μg), Ciprofloxacin (5 μg), levofloxacin (5 μg), Trimethoprim/ sulfamethoxazole (1.25/23.25 μg), gentamicin (10 μg), Vancomycin (30 μg) and Linezolid (30 μg).

### Statistical analysis

Data obtained were double entered into a spreadsheet database prepared with Microsoft Excel and compared and cleaned for wrong entries. Statistical analysis was done using SAS version 9.1 [20]. The association of each of the categorical variables with the response variable was assessed by Chi-square test. Further, logistic regression was performed, and variables showing statistically significant (*p* < 0.05) association in univariate analysis with the outcome variable were considered following multivariate analysis as a risk factor. Further, 2 way-ANOVA was performed to assess the significance of PCT between symptomatic and asymptomatic CLABSI. Continuous variables were expressed as a measure of central tendency.

## Results

Over the 3 years, 1462 children (2 to 12 years of age) who are newly undergoing long-term HD were included. The mean age of the participant was 7.82 ± 2.62 years. Children with congenital malformations and obstructive uropathy requiring HD was significant among children below the age of 5 years (*n* = 642, mean age 2.82 ± 1.43 years, *p* = 0.03) while hereditary kidney diseases requiring HD was significant in the age group of 5 to 12 years (*n* = 822, mean age 8.82 ± 1.22, *p* = 0.01).

### Blood culture results

Blood cultures were positive in 1090 (74.5%) out of 1462 children on long term HD. According to differential time positivity, 410 (28%) were having CLABSI while 520 (35.6%) were having bacteremia without CLABSI, and 160 (11%) were having central venous line colonizers. Remaining 372 (25.4%) was culture negative.

#### CLABSI

Out of 410 CLABSI patients 331 (80.7%) were symptomatic. They were presented with fever (*n* = 331), malaise (*n* = 331), and 58 (17.5%) were septic (fever, tachypnoea, tachycardic, PCT > 0.5 with serum lactate > 4 mmol/L) on admission. Methicillin sensitive *Staphylococcus aureus* (MSSA) was the commonest etiology in symptomatic CLABSI and it was isolated in 212 (64.7%, *p* = 0.02) patients. *Candida albicans* (*n* = 17,4.5%) *C. parapsilosis* (*n* = 10, 3.1%) and Gram negatives [multi-drug resistant *Klebsiella pneumoniae* (*n* = 10, 3.1%) *Escherichia coli* (*n* = 6, 1.5%) and *Citrobacter frundii* (*n* = 3, 0.75%)] were also isolated.

**Fig. 1:**
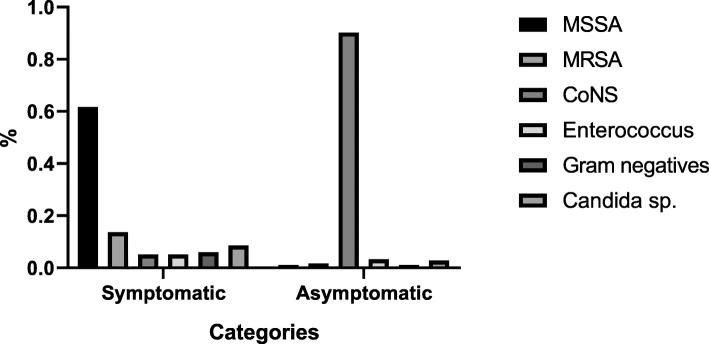
Microbial etiology in symptomatic and symptomatic patients with central line-associated bloodstream infections. MSSA- Methicillin-sensitive *S. aureus*, MRSA- Methicillin-resistant *S. aureus*, CoNS- Coagulase-negative *Staphylococcus sp.*

Out of 410 CLABSI patients 79 (19.3%) were asymptomatic. Coagulase negative staphylococcus sp. [*S. epidermidis* (*n* = 54, 68.4%), *S. hemolyticus* (*n* = 6, 7.7%), *S. schleiferi* (*n* = 5, 6.4%), *S. lugdunensis* (*n* = 5, 6.4%) (*p* = 0.03)] was the commonest etiology for asymptomatic CLABSI (Fig. 1).

Recurrent bacteremia was detected in both symptomatic (*n* = 98, 23.9%, *P* = 0.02) and asymptomatic CLABSI groups while it was significant in the former. MSSA was the most frequent etiology in recurrent symptomatic CLABSI, and it was isolated in 78 (79.5%, *p* = 0.01) patients.

### Bacteremia without CLABSI

Out of 520 bacteremic patients 398 (76.5%) were symptomatic. They were presented with fever (*n* = 398), malaise (*n* = 398), cough (*n* = 90), difficulty in breathing (*n* = 88), dysuria (*n* = 48), backache (*n* = 22), acute abdomen (*n* = 10), alteration of consciousness (*n* = 8), and 68 (17.1%) were septic (fever, tachypnoea, tachycardic, PCT > 0.5 with serum lactate > 4 mmol/L) on admission. Methicillin sensitive *S. aureus* (MSSA) was the commonest etiology and was isolated in 245 (61.5%, *p* = 0.03) patients. Methicillin resistant *S. aureus* (MRSA) in 42 (10.5%) patients while coagulase negative staphylococcus sp.(45, 11.3%) [*S. epidermidis* (*n* = 27,6.8%), S. hemolyticus (*n* = 18, 4.5%))]*, E. fecalis* (*n* = 8, 2%), *E. faceum* (*n* = 4, 1%), *Candida albicans* (*n* = 8,2%) *C. parapsilosis* (*n* = 8, 2%), *C. tropicalis* (*n* = 7,2%) and *K. pneumoniae* (*n* = 10, 2.5%) *E. coli* (*n* = 16, 4%), *Pseudomonas aeruginosa* (*n* = 8, 4%), and *C. frundii* (*n* = 6, 3%) was also isolated.

**Fig. 2:**
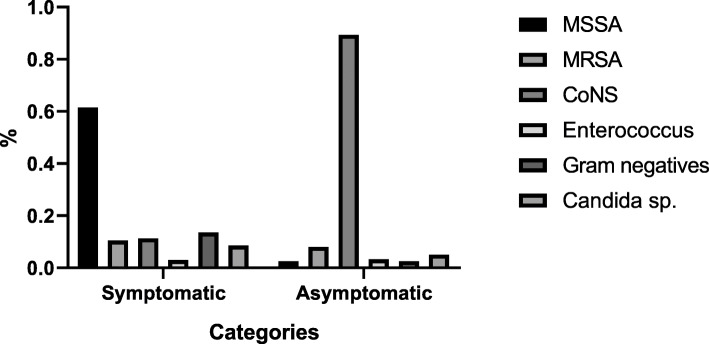
Microbial etiology in symptomatic and symptomatic bacteremic patients. MSSA- Methicillin-sensitive *S. aureus*, MRSA- Methicillin-resistant *S. aureus*, CoNS- Coagulase-negative *Staphylococcus sp.*

Out of 520 bacteremic patients 122 (23.4%) were asymptomatic. Coagulase negative staphylococcus sp. [*S. epidermidis* (*n* = 86, 70.5%), *S. hemolyticus* (*n* = 14, 11.4%), *S. schleiferi* (*n* = 6, 5%), *S. lugdunensis* (*n* = 3, 2.5%) (*p* = 0.01)] was the commonest etiology for asymptomatic bacteremic and *C. parapsilosis* (*n* = 3, 2.5%), *C. albicans* (*n* = 3, 2.5%) MSSA (*n* = 3, 2.5%) MRSA (1,0.8%), *Escherichia coli* (*n* = 3, 2.5%) was also isolated (Fig. 2).

Recurrent bacteremia was detected in both symptomatic (*n* = 78, 19.6%, *P* = 0.04) and asymptomatic groups while it was significant in the former. MSSA was the most frequent etiology in recurrent symptomatic bacteremia, and it was isolated in 58 (74.3%, *p* = 0.03) patients.

### Venous catheter colonizer (VCC)

Out of 160 VCC, coagulase negative *Staphylococcus* sp. [*S. epidermidis* (*n* = 86, 53.7%), *S. hemolyticus* (*n* = 16, 10%), *S. schleiferi* (*n* = 8, 5%), *S. lugdunensis* (*n* = 4, 2.5%) (*p* = 0.01)] was the commonest while *C. parapsilosis* (*n* = 12, 7.5%), *C. albicans* (*n* = 8, 5%), MSSA (*n* = 8, 5%) MRSA (4,2.5%), *Escherichia coli* (*n* = 4, 2.5%), *Acinetobacter bahumanii* (*n* = 4, 2.5%), *C. frundii* (*n* = 4, 2.5%) and *P. aeruginosa* (*n* = 4, 2.5%) was also isolated.

In all patients with bacteremia, procalcitonin was elevated (> 0.5 ng/ml). PCT was in asymptomatic CLABSI was 1.8 ± 0.9 while in symptomatic CLABSI was 11.3 ± 2.5 (*p* = 0.01).

### Antimicrobial susceptibility pattern of isolated microbes from asymptomatic and symptomatic CLABSI

Compared to *S. epidermidis* isolates following symptomatic CLABSI, *S. epidermidis* isolates following asymptomatic CLABSI were having significant resistance to oxacillin, cefoxitin, ciprofloxacin, levofloxacin, Trimethoprim/ sulfamethoxazole, Amikacin, gentamicin, erythromycin, and clindamycin while all were susceptible to vancomycin, linezolid, and tigecycline (Table 1).

**Table 1:** Antimicrobial susceptibility profile of coagulase-negative *Staphylococcus* sp. (CoNS) isolates in asymptomatic and symptomatic central line-associated bloodstream infections

Antibiotic	CLABSI						***P value***
	Symptomatic- CoNS		Asymptomatic- CoNS				
	***S. epidermidis*** (***n*** = 10)	***S. hemolyticus*** (***n*** = 6)	***S. epidermidis*** (***n*** = 54)	***S. hemolyticus*** (***n*** = 6)	*S. schleiferi* (***n*** = 5)	***S. lugdunensis*** (***n*** = 5)	
**Ampicillin (30 μg)**	2 (20%)	1 (17%)	47 (87.3%) ^*^	6 (100%)	5 (100%)	5 (100%)	0.03^*^
**Cefoxitin (30 μg)**	2 (20%)	1 (17%)	45 (85.7%) ^**^	6 (100%)	5 (100%)	5 (100%)	0.02^**^
**Oxacillin**	2 (20%)	1 (17%)	45 (85.7%) ^***^	6 (100%)	5 (100%)	5 (100%)	0.03^***^
**Ciprofloxacin (5 μg)**	1 (10%)	1 (17%)	45 (85.7%) ^α^	4 (67%)	5 (100%)	5 (100%)	0.03^α^
**Levofloxacin (5 μg)**	2 (20%)	1 (17%)	45 (85.7%) ^**μ**^	4 (67%)	4 (80%)	5 (100%)	0.03^μ^
**Trimethoprim/ sulfamethoxazole (1.25/23.25 μg)**	1 (10%)	1 (17%)	42 (79.3%)	4 (67%)	5 (100%)	5 (100%)	0.03
**Amikacin (30 μg)**	3 (30)	1 (17%)	42 (79.3%)	4 (67%)	5 (100%)	5 (100%)	0.03
**Gentamicin (10 μg)**	2 (20)	1 (17%)	42 (79.3%)	4 (67%)	5 (100%)	5 (100%)	0.03
**Erythromycin (30 μg)**	2 (20)	1 (17%)	42 (79.3%)	4 (67%)	5 (100%)	5 (100%)	0.03
**Clindamycin (30 μg)**	2 (20)	1 (17%)	42 (79.3%)	4 (67%)	5 (100%)	5 (100%)	0.03
**vancomycin**	**0**	**0**	**0**	**0**	**0**	**0**	**–**
**Linezolid**	**0**	**0**	**0**	**0**	**0**	**0**	**–**
**Tigecycline**	**0**	**0**	**0**	**0**	**0**	**0**	**–**

*P* < 0.05 taken as significant

* Ampicillin resistance is significantly low in S. epidermidis; ** Cefoxitin resistance is significantly low in S. epidermidis; *** Oxacillin resistance is significantly low in S. epidermidis; α Ciprofloxacin resistance is significantly low in S. epidermidis; μ Levofloxacin resistance is significantly low in S. epidermidis

### Other infective complications

Central venous catheter exit site (*n* = 98, 6.7%) and tunnel infections (*n* = 70, 4.8%) were detected in long term indwelling venous catheters. Exit site infection was observed 7.5 ± 6.25 months, and tunnel infection was observed 11 ± 2.75 months following the insertion of a venous catheter. Further, venous catheter blockage was detected in 88 (6%), and in all, including exit and tunnel infections catheter, revisal was done to minimize systemic infections. The blockage was observed 0.82 ± 0.43 years following the insertion of a central venous catheter.

Right sided infective endocarditis (IE) was detected in 122 (8.3%) patients and was observed 8.6 ± 3 months following insertion of venous catheter. It was detected in CLABSI symptomatic (*n* = 29, 24.5%), CLABSI asymptomatic (*n* = 42, 34.5%)^,^ symptomatic (*n* = 14, 11.5%) and asymptomatic bacteremic (*n* = 36, 29.5%) patients and is significant in asymptomatic CLABSI and asymptomatic bacteremic patients (Table 2).

**Table 2:** The microbial etiology for right-sided infective endocarditis in symptomatic and asymptomatic central line-associated bloodstream infections and bacteremia without central line infection patients

Isolated microbes	Right sided infective endocarditis (***n*** = 122, 8.3%)				***P*** value
	CLABSI (72, 59%)		Bacteremia without CLABSI (50, 41%)		
	Symptomatic (***n*** = 30, 24.5%)	Asymptomatic (***n*** = 42, 34.5%)	Symptomatic (***n*** = 14, 11.5%)	Asymptomatic (***n*** = 36, 29.5%)	0.03
**MSSA**	12^**^	13^**^	4	6	0.03^**^
**MRSA**	12^£^	13^£^	7^£^	7	0.02^£^
**CoNS**	2	14^α^	1	16^α^	0.02^α^
*S. epidermidis*	1	6	–	9	0.07
*S. hemolyticus*	1	1	1	2	0.08
*S. schleiferi*	–	1	–	4	0.07
*S. lugdunensis*	–	1	–	1	0.07
Enterococcus sp.	2	1	–	1	0.08
*C. albicans*	3	1	1	2	0.07
*C. parapsilosis*	1	1	1	4	0.08

*CLABSI* central line-associated bloodstream infections, *MSSA* Methicillin-sensitive *S. aureus*, *MRSA* Methicillin-resistant *S. aureus*, *CoNS* Coagulase-negative *Staphylococcus sp.*

*P* < 0.05 taken as significant

^**^ MSSA was associated significantly in symptomatic and asymptomatic CLABSI, ^£^MRSA was associated significantly in right sided IE following symptomatic, asymptomatic CLABSI and symptomatic bacteremic without CLABSI and ^α^ CoNS was associated significantly in right sided IE following asymptomatic CLABSI and asymptomatic bacteremic without CLABSI

Further, catheter tip endocarditis (*n* = 88, 6%) and left sided IE (*n* = 31, 2.1%) was detected in all above mentioned groups. Catheter tip endocarditis was observed 6.25 ± 2.9 months following insertion of venous catheter and it was significantly detected in asymptomatic bacteremic (without CLABSI) (*n* = 48, 54.5%, *p* = 0.03) and non bacteremic children (*n* = 39, 44.3%, *p* = 0.04). Coagulase negative *Staphylococcus sp.[s. epidermidis* (*n* = 18, 37.5%), *S. hemolyticus* (*n* = 12, 25%), *S. schleiferi* (*n* = 6, 12.5%), *S. lugdunensis* (*n* = 3, 6.25%) (*p* = 0.01)] was the commonest while *C. parapsilosis* (*n* = 4, 8.3%), *C. albicans* (*n* = 3, 6.25%), MSSA (*n* = 1, 1.1%) and MRSA (*n* = 1, 1.1%) also isolated from the blood cultures.

Patients with left sided IE was observed 0.42 ± 0.34 years following insertion of venous catheter and it was detected in symptomatic bacteremic (*n* = 19, 61.2%, *p* = 0.03) and symptomatic CLABSI children (*n* = 12, 38.7%, *p* = 0.04). MRSA in 12 (38.7%), MSSA (*n* = 10, 34%), *E. fecalis* (*n* = 3, 10%), *E. faceum* (*n* = 3, 10%), *C. albicans* (*n* = 2, 6.7%) and *C. parapsilosis* (*n* = 1, 3.3%) was detected in patients with left sided IE.

Following univariate analysis, coagulase-negative *Staphylococcus* sp., tunneled central venous catheter, PICC, femoral site, a number of line-days > 90, receipt of vancomycin, meropenem or linezolid in the 5 days before obtaining blood cultures and recurrent bacteremia was significantly associated with increased risk of asymptomatic CLABSI. Further, receipt of vancomycin, meropenem, or linezolid in the 5 days before obtaining blood cultures was significantly associated with non-bacteremia (Table 3).

**Table 3:** Univariate analysis of risk factors for asymptomatic central line-associated bloodstream infections in children on hemodialysis

Variables	CLABSI (***n*** = 410)		Non-bacteremics Odds ratio (95% CI)
	Symptomatic Odds ratio (95% CI)	Asymptomatic Odds ratio (95% CI)	
**Microbial etiology**			
**MSSA**	**2.2 (1.1–3.9)**		
**MRSA**	**2.4 (1.9–3.1)**		
**CoNS**		**3.4 (2.9–3.8)**	
**Female sex**	–	–	
**Type of CVC**			
**Tunneled**		**2.7 (1.8–4.3)**	
**Non-tunneled**	–	–	–
**Site of insertion**			
**Internal jugular**	–	–	–
**PICC**		**2.9 (1.9–4.2)**	
**Subclavian**	–	–	–
**Femoral**		**2.7 (1.8–4.3)**	
**Number of line-days > 90**		**2.3 (1.3–4.0)**	
**Duration of hospital stay**	–	–	–
**Receipt of vancomycin, meropenem or linezolid in the 5 days prior to obtaining blood cultures**		**3.7 (2.8–4.6)**	**4.7 (3.8–5.3)**
**No. of times intravenous medications given in the prior day (mean ± SD)**	–	–	–
**Recurrent bacteremia**		**2.7 (1.8–4.3)**	

All significant (*P* < 0.05) values are displayed with the odds ratio

*CLABSI* central line-associated bloodstream infections, *MSSA* Methicillin-sensitive *S. aureus*, *MRSA* Methicillin-resistant *S. aureus*, *CoNS* Coagulase-negative *Staphylococcus sp., CVC* central venous catheter, *PICC* peripherally inserted central catheter, *SD* standard deviation and *CI* confidence interval

According to multivariate analysis coagulase negative staphylococcus sp. blood stream infection (OR: 7.6, 95% CI: 6.5–8.3, *P* = 0.03)**,** having tunneled CVC (OR: 4.4, 95% CI: 3.8–4.9, *P* = 0.03), peripherally inserted central catheter (OR: 4.9, 95% CI: 3.9–6.2, *P* = 0.02), femoral site (OR: 3.7, 95% CI: 2.8–4.3, *P* = 0.01), number of line days > 90 (OR: 2.1, 95% CI: 1.3–4.0, *P* = 0.02), receipt of vancomycin, meropenem or linezolid in the 5 days prior to CLABSI diagnosis (OR: 2.1, 95% CI: 1.3–4.0, *P* = 0.02) and recurrent bacteremia (OR: 8.7, 95% CI: 7.8–9.3, *P* = 0.01) were associated with asymptomatic CLABSI in patients with long term hemodialysis (Table 4).

**Table 4:** Multivariate analysis of risk factors for asymptomatic central line-associated bloodstream infections in children on hemodialysis

Variables	Asymptomatic CLABSI Odds ratio (95% CI) P value	
**CoNS bloodstream infection**	7.6 (6.5–8.3)	0.01
**Tunneled-CVC**	4.4 (3.8–4.9)	0.03
**Site of insertion -PICC**	4.9 (3.9–6.2)	0.02
**Site of insertion -Femoral**	3.7 (2.8–4.3)	0.01
**Number of line-days > 90**	2.1 (1.3–4.0)	0.02
**Receipt of vancomycin, meropenem or linezolid in the 5 days prior to CLABSI diagnosis**	3.7 (2.8–4.6)	0.01
**Recurrent bacteremia**	8.7 (7.8–9.3)	0.01

*CLABSI* central line-associated bloodstream infections, *MSSA* Methicillin-sensitive *S. aureus*, *MRSA* Methicillin-resistant *S. aureus*, *CoNS* Coagulase-negative *Staphylococcus sp., CVC* central venous catheter, *PICC* peripherally inserted central catheter and CI- confidence interval

*P* < 0.05 taken as significant

## Discussion

CVCs are not without risk and following placement it can develop multiple complications. Significant morbidity, mortality can result following complications and can cause a significant burden leading to high expenditure, prolonged hospitalization, and poor quality of life [21–23].

To our knowledge, this is the first report of asymptomatic CLABSI in patients who are undergoing long term HD. Transient asymptomatic bacteremia is a known phenomenon, while CLABSI is often symptomatic and followed with continuous bacteremia [5, 6]. Contrary, asymptomatic CLABSI could be a rare occurrence, and we were able to explore it following screening blood cultures in every patient before each HD. CoNS was predominantly isolated in patients with asymptomatic CLABSI, while MSSA was predominantly isolated in patients with symptomatic CLABSI. Zierdt in 1983 has described that the intermittent or transient or asymptomatic *S. epidermidis* bacteremia can frequently occur inpatients and as well as in healthy humans [24].

Right-sided IE is a well-known complication following long term indwelling CVCs [15]. Often is diagnosed with screening echocardiography following MSSA, MRSA bacteremia or candidemia, and instances where having an inadequate response following appropriate antimicrobials in other bacteremia (CoNS, *Enterococcus* sp., any of significant bacteremia) [22–25]. In here, CoNS was significantly associated with right-sided IE following asymptomatic CLABSI and asymptomatic bacteremia without CLABSI patients. Kendirli et al. in 2017 found that *S. epidermidis* was commonly identified in children with CLABSI [26]. Another study revealed that *S. aureus* was the most common organism responsible for right sided IE and IE in tricuspid valve develop more frequently in heroin users [27]. Further, MSSA or MRSA, *Streptococci* spp. or *Enterococci* spp. is responsible for acute IE [28]. In here, MSSA was associated significantly following right sided-IE in symptomatic and asymptomatic CLABSI patients while MRSA was associated significantly in right-sided IE following symptomatic, asymptomatic CLABSI and symptomatic bacteremia without CLABSI patients. The above scenarios were detected as an outcome of screening blood cultures and echocardiography. Application of such practice in busy and resource-limited clinical setup needs to be validated.

Contrast to *S. epidermidis* following symptomatic CLABSI, *S. epidermidis* following asymptomatic CLABSI had significant antimicrobial resistance. It could be an outcome following the segregation of multiple genetic segments in microbial DNA. Since bacterial genetic capacity is often constant, this would reduce the virulence capacity. Various studies have shown that the genetic perturbations responsible for antibiotic resistance modulate bacterial biology and fitness [29]. In many cases, measurements of bacterial growth in animal hosts have revealed fitness and virulence attenuations that agree with in vitro tests, leading to the view that pathogens incur fitness trade-offs that compromise their pathogenic potential. Perhaps, the drug resistance increases pathogen fitness during infection [30, 31].

Based on multivariate analysis having a CoNS bloodstream infection and tunneled CVC were risk factors for the development of asymptomatic CLABSI in patients with long term HD. Having tunneled CVC is a known risk factor in CLABSI [32]. Further, the peripherally inserted central catheter (PICC) and femoral site were risk factors for the development of asymptomatic CLABSI in patients with long term HD. Both PICC [33] and femoral site [34] is known risk factor in CLABSI. Contrary, no evidence of higher infection risk when the catheter is inserted into the subclavian, jugular, or femoral vein, as demonstrated for adult patients [33]. A number of line days > 90 was a risk factor for the development of asymptomatic CLABSI in patients with long term HD. The risk of bacteremia is highest in hemodialysis patients using a CVC for vascular access, and increases in a linear fashion with the duration of catheter use [2, 4–8]. Prolonged use of CVC (7 or more days) considered the risk for CLABSI [31]. A study by Costello et al. analyzed 3319 admissions to the pediatric cardiac intensive care unit found that a central venous line in place for ≥7 days [34]. Also, receipt of vancomycin, meropenem, or linezolid in the 5 days before CLABSI diagnosis was a risk factor for the development of asymptomatic CLABSI in patients with long term hemodialysis. Receipt of high-end antibiotics (vancomycin, meropenem, or linezolid) would suppress the symptoms of bacteremia in great. Because the use of such broad-spectrum antibiotics around 5 days would suppress the bacteremia leading state of partially treated [35]. Depending on etiology for bacteremia without detected focus, at least 7–14 days of therapy is recommended [28]. Furthermore, recurrent bacteremia was a risk factor for the development of asymptomatic CLABSI in patients with long term hemodialysis. Recurrent bacteremia is a risk factor for the development of CLABSI [36, 37].

Here, PCT was in asymptomatic CLABSI was 1.8 ± 0.9 ng/mL while in symptomatic CLABSI was 11.3 ± 2.5 ng/mL. The procalcitonin cut-off value to detect sepsis was ≥0.5 ng/mL, with a corresponding sensitivity of 76% and specificity of 69%. Different studies done in different settings the procalcitonin had a fair diagnostic accuracy for bacteremia in adult patients suspected of infection or sepsis. Perhaps, low procalcitonin levels can be used to rule out the presence of bacteremia [38, 39].

### Limitations

We have not performed anaerobic blood cultures to detect any of anaerobic etiology. Specific fungal cultures using a semi-automated platform was also not performed. We have used only the DTP method to detect CLABSI. The overall sensitivity and specificity of a DTP of ≥120 min for diagnosing CLABSI were 85% (95% confidence interval [CI], 74 to 93%) and 82% (95% CI, 66 to 92%), respectively. Further, we have not performed *S. epidermidis* molecular genetics related to antimicrobial resistance. Due to the low number of MSSA and MRSA cases, risk factor analysis was unable to perform related to symptomatic and asymptomatic CLABSI.

## Conclusions

Asymptomatic CLABSI could be a rare occurrence. CoNS was predominantly isolated in patients with asymptomatic CLABSI. Right-sided IE is a well-known complication following long term indwelling central venous catheters. CoNS was significantly associated with right-sided IE following asymptomatic CLABSI and asymptomatic bacteremia without CLABSI patients. Regular procalcitonin, microbiological, and imaging studies will be essential to detect infectious complications in both symptomatic and asymptomatic children implanted with long term indwelling CVCs.

## Data Availability

The datasets used and analyzed in the current study are available from the corresponding author on reasonable request.

## Abbreviations

CVC: Central venous catheter
CLABSI: Central line-associated bloodstream infection
USA: United State of America
PICC: Peripherally inserted central catheter
HD: Hemodialysis
CoNS: Coagulase-negative *Staphylococcus sp.*
RS-IE: Right-sided infective endocarditis
CKD: Chronic Kidney disease
DTP: Differential time positivity
PCT: Procalcitonin
MRSA: Methicillin-resistant *Staphylococcus aureus*
MSSA: Methicillin sensitive *Staphylococcus aureus*
CI: Confidence interval
